# Structure of hyperthermophilic β-glucosidase from *Pyrococcus furiosus*

**DOI:** 10.1107/S1744309111035238

**Published:** 2011-11-25

**Authors:** Yuji Kado, Tsuyoshi Inoue, Kazuhiko Ishikawa

**Affiliations:** a Health Research Institute, National Institute of Advanced Industrial Science and Technology (AIST), 1-8-31 Midorigaoka, Ikeda, Osaka 563-8577, Japan; b Division of Applied Chemistry, Graduate School of Engineering, Osaka University, 2-1 Yamadaoka, Suita, Osaka 565-0871, Japan; c Biomass Technology Research Center, National Institute of Advanced Industrial Science and Technology (AIST), 3-11-32 Kagamiyama, Higashi-Hiroshima, Hiroshima 739-0046, Japan

**Keywords:** hyperthermophilic, cellulases, biomass, *Pyrococcus furiosus*

## Abstract

Three categories of cellulases, endoglucanases, cellobiohydrolases and β-glucosidases, are commonly used in the process of cellulose saccharification. In particular, the activity and characteristics of hyperthermophilic β-glucosidase make it promising in industrial applications of biomass. In this paper, the crystal structure of the hyperthermophilic β-glucosidase from *Pyrococcus furiosus* (BGLPf) was determined at 2.35 Å resolution in a new crystal form. The structure showed that there is one tetramer in the asymmetric unit and that the dimeric molecule exhibits a structure that is stable towards sodium dodecyl sulfate (SDS). The dimeric molecule migrated in reducing SDS polyacrylamide gel electrophoresis (SDS–PAGE) buffer even after boiling at 368 K. Energy calculations demonstrated that one of the two dimer interfaces acquired the largest solvation free energy. Structural comparison and sequence alignment with mesophilic β-glucosidase A from *Clostridium cellulovorans* (BGLACc) revealed that the elongation at the C-terminal end forms a hydrophobic patch at the dimer interface that might contribute to hyperthermostability.

## Abstract 2

Recombinant hyperthermophilic β-glucosidase from *P. furiosus* was crystallized. The crystal structure was solved to a resolution of 2.35 Å.

## 1. Introduction   

Cellulosic materials constitute most of the biomass on Earth and are capable of being converted into bioethanol, a next-generation biofuel (Bayer & Lamed, 1992 ▶; Farrell *et al.*, 2006 ▶; Joshi & Mansfield, 2007 ▶; Ragauskas *et al.*, 2006 ▶). The process of bioethanol production from biomass requires the saccharification of cellulose in order to obtain fermentable sugars. In nature, cellulolytic microbes typically produce three categories of cellulases which convert cellulose into glucose: endoglucanases (EGs), cellobiohydrolases (CBHs) and β-glucosidases (BGLs) (Baldrian & Valásková, 2008 ▶; Stricker *et al.*, 2008 ▶; Tomme *et al.*, 1995 ▶). Cellulase systems using these three types of enzymes show potential for complete industrial-scale enzymatic saccharification of cellulose. In this setting, *Trichoderma reesei* has been considered to be a strongly cellulolytic and xylanolytic candidate microorganism. However, complete saccharification of cellulose is not accomplished by the cellulases isolated from *T. reesei* because its BGL exhibits low activity. To overcome this problem, BGL from *Aspergillus aculeatus* (BGLAa) has been used to increase the cellulase activity of *T. reesei* (Kawaguchi *et al.*, 1996 ▶).

The hyperthermophilic β-glucosidase from *Pyrococcus furiosus* (BGLPf) belongs to the glycoside hydrolase 1 (GH1) family. The enzymes of this family form (β/α)_8_ barrels and hydrolyze their substrate while retaining configuration at the anomeric C atom. Two glutamate residues serve as a general acid/base or nucleophile in the reaction. A site-directed mutagenesis approach revealed that the catalytic dyad of BGLPf, composed of Glu207 (acid/base) and Glu372 (nucleophile), hydrolyzes the β-1,4 bonds of its substrates (Voorhorst *et al.*, 1995 ▶).

To date, a thermophilic cellulase system for industrial conversion of biomass has not been developed. Nevertheless, enzymatic degradation of biomass at high temperature would provide obvious advantages, such as limiting bacterial contamination and increasing substrate solubility. Recently, an endocellulase (EGPh, family 5) from the hyperthermophilic archaeon *P. horikoshii* was identified and recombinant EGPh was successfully expressed using *Escherichia coli* (Ando *et al.*, 2002 ▶; Kashima *et al.*, 2005 ▶; Kim *et al.*, 2007 ▶, 2008 ▶). EGPh exhibits progressive hydrolytic activity, releasing cellobiose after an initial endo-type attack on cellulose. Hyperthermophilic archaeal BGLs have also been isolated from *P. horikoshii* and *P. furiosus* (Lebbink *et al.*, 2001 ▶; Matsui *et al.*, 2000 ▶). BGL from *P. horikoshii* (BGLPh) exhibits specific activity towards cellobiose, but not towards other cellooligosaccharides (Matsui *et al.*, 2000 ▶). Furthermore, the activity of BGLPh was only observed in the presence of detergents (Matsui *et al.*, 2000 ▶). In contrast, BGL from *P. furiosus* (BGLPf) exhibits specific activity towards a wide range of substrates, but its highest hydrolytic activity is towards cellooligosaccharides at high temperature (Kaper *et al.*, 2000 ▶; Bauer *et al.*, 1996 ▶). The activity and substrate specificity of BGLPf (Kim & Ishikawa, 2010 ▶) make it a candidate enzyme for the saccharification of biomass.

31 structures in the GH1 family have been reported to date. The crystal structure of BGLPf has also been determined to a resolution of 3.3 Å (Kaper *et al.*, 2000 ▶). However, a structural model has not been built and detailed information about the structure of this enzyme is not available from the low-resolution data set. Moreover, structural data regarding BGLPf have not been deposited in the Protein Data Bank (PDB). Here, the structure of a new crystal form of BGLPf was determined to a resolution of 2.35 Å. The crystal structure was examined to reveal information on the hyperthermo­stability and the substrate-recognition mechanism of BGLPf.

## 2. Materials and methods   

### 2.1. Protein preparation   

BGLPf (Gene ID PF0073; Bauer *et al.*, 1996 ▶) was purified as follows. The recombinant protein was expressed in *Escherichia coli* BL21 (DE3) cells (Novagen) under control of the T7 promoter in pET11a (Novagen). Cell cultures were grown at 310 K in Luria broth (3.2 l) containing 100 µg ml^−1^ ampicillin until the optical density at 600 nm (OD_600_) reached 0.8. Isopropyl β-d-1-thiogalactopyranoside was added to a final concentration of 1.0 m*M* for protein induction. The harvested cells were lysed by sonication in 50 m*M* Tris–HCl pH 8.0 at 277 K. The cell lysate was heat-treated at 358 K for 30 min and then centrifuged at 15 000*g* for 20 min at 277 K. Streptomycin (2 g) was added to the supernatant (100 ml) at 277 K with stirring and the mixture was centrifuged at 15 000*g* for 30 min. The supernatant was fractionated with ammonium sulfate up to 80% saturation. After centrifugation, the pellet was resuspended in 50 m*M* Tris–HCl pH 8.0 and then dialyzed against Tris–HCl pH 8.0. The lysate was loaded onto a HiTrap Q anion-exchange column (GE Healthcare Bio­sciences) equilibrated with 50 m*M* Tris–HCl pH 8.0 and eluted with a linear gradient of 0–0.5 *M* NaCl. The composition of the buffer solution containing the target sample was adjusted to 50 m*M* Tris–HCl pH 8.0 containing 20%(*v*/*v*) ammonium sulfate. This solution was loaded onto a hydrophobic HiTrap Phenyl column (GE Healthcare Biosciences) equilibrated with 20 m*M* Tris–HCl buffer pH 8.0 containing 20%(*v*/*v*) ammonium sulfate and was eluted with a linear gradient of 20–0% ammonium sulfate. The purity and the size of the protein were analyzed by reducing SDS–PAGE. The size of the enzyme oligomer was examined by gel filtration using Hi-Load 26/60 Superdex 200 pg (GE Healthcare Biosciences). The con­centration of BGLPf was determined from the UV absorbance at 280 nm using a molar extinction coefficient of 128 160 *M* ^−1^ cm^−1^ as calculated from its protein sequence using a standard method (Gill & von Hippel, 1989 ▶).

### 2.2. Crystallization   

Some crystals were obtained using the conditions described by Kaper *et al.* (2000 ▶), but their quality was too poor to allow X-ray analysis. Thus, initial screening for optimal crystallization conditions was performed using Crystal Screen, Crystal Screen 2 (Hampton Research) and Wizard 1 and 2 (Emerald BioSystems) with the hanging-drop vapour-diffusion method at 293 K. Typically, drops consisting of 1 µl protein solution (10 mg ml^−1^ in 20 m*M* Tris–HCl pH 8.0) and 1 µl reservoir solution (0.1 *M* Na HEPES pH 7.5 con­taining 0.8 *M* sodium phosphate monobasic monohydrate and 0.8 *M* potassium phosphate monobasic) were equilibrated against 0.4 ml reservoir solution. A crystal was obtained within one week at 293 K.

### 2.3. Data collection and processing   

The selected crystal was immersed in a cryoprotectant consisting of 25%(*v*/*v*) glycerol solution, picked up in a loop and then flash-cooled in a stream of nitrogen gas at 100 K. X-ray diffraction data were collected using a Rayonix MX225HE detector at a wavelength of 0.9 Å on the BL41XU beamline at SPring-8 (Hyogo, Japan). The crystal-to-detector distance was 300 mm. The crystal was rotated through 180° with an oscillation angle of 0.5° per frame. The data collected from diffraction measurements were indexed, integrated and scaled with programs from the *HKL*-2000 software package (Otwinowski & Minor, 1997 ▶). Diffraction data were collected to a resolution of 2.35 Å. Data-collection and processing parameters are presented in Table 1 ▶.

### 2.4. Structure solution and refinement   

The structure was solved by molecular replacement with *MOLREP* (Vagin & Teplyakov, 2010 ▶) using the structural data for the BGL monomer from *Thermosphaera aggregans* (BGLTa; 61% sequence identity to BGLPf; PDB entry 1qvb; Chi *et al.*, 1999 ▶) as the search model. Further iterations of refinement and model building were performed with *REFMAC*5 (Murshudov *et al.*, 2011 ▶), *CNS* (Brünger *et al.*, 1998 ▶) and *Coot* (Emsley & Cowtan, 2004 ▶). Noncrystallographic symmetry (NCS) restraints were not applied during the refinement. The presence of four enzyme molecules per asymmetric unit gave a crystal volume per protein mass (*V*_M_) of 3.96 Å^3^ Da^−1^ and a solvent content of 69%(*v*/*v*) (Matthews, 1968 ▶). The quality of the refined structure was checked with *MolProbity* (Chen *et al.*, 2010 ▶). A Ramachandran plot showed that 96.7% of residues were in the favoured regions and 99.6% were in allowed regions. The structural data have been deposited in the PDB under accession code 3apg.

## 3. Results and discussion   

### 3.1. Crystal structure of the tetrameric form of BGLPf   

**Figure 1:**
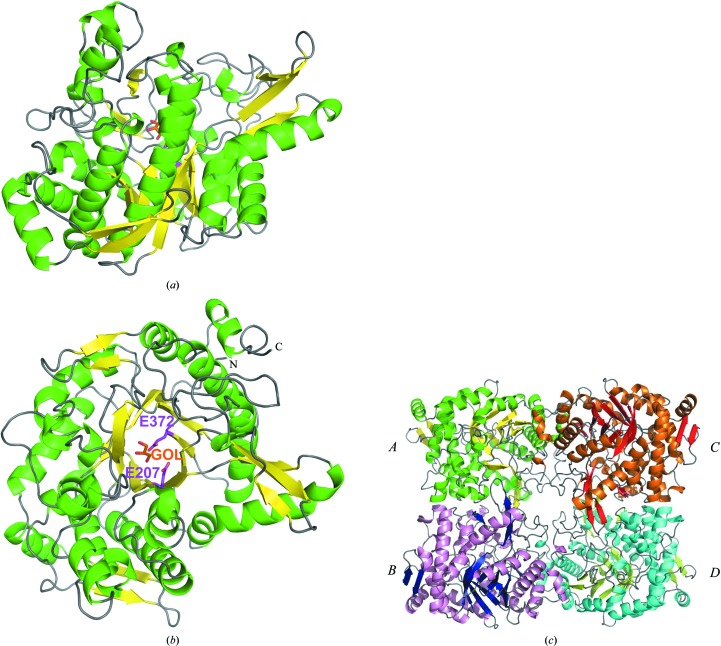
Overall structure of BGLPf. (*a*) Side view, (*b*) top view. The catalytic dyad (Glu207, Glu372) and a glycerol molecule are shown in purple and red, respectively. (*c*) Structure of a tetramer of BGLPf.

A tetrameric structure was identified in the crystallographic asymmetric unit of BGLPf and was determined to a resolution of 2.35 Å (Table 1 ▶). The tetramer shows 222 point-group symmetry and each monomer contacts all three symmetry-related partners. The monomer model contains 471 amino-acid residues and a (β/α)_8_ barrel fold (Figs. 1 ▶ *a* and 1 ▶ *b*). The active site is located at the centre of the monomer and is reached from the outside by a tunnel with a length of 20 Å. A molecule of glycerol, which was used as cryoprotectant, was observed in the active site of each of the four monomers. Based on a comparison between the structures, a root-mean-square deviation (r.m.s.d.) value of 0.57 Å for 417 C^α^ atoms was calculated between BGLPf and BGLTa.

The individual monomers in the tetramer structure were named *A*, *B*, *C* and *D* (Fig. 1 ▶ *c*). The structure of monomer *A* in BGLPf was compared with those of *B*, *C* and *D*, with r.m.s.d. values ranging from 0.15 to 0.20 Å over 469–470 C^α^ atoms. A similar structure consisting of homotetramers has previously been reported in another crystal form determined at 3.3 Å resolution (Kaper *et al.*, 2000 ▶).

Gel filtration of BGLPf gave a single peak from which the molecular weight of the protein was estimated to be 238.8 kDa, which is similar to that of the BGLPf tetramer (220 kDa), suggesting that BGLPf predominantly forms tetramers (Supplementary Fig. 1^1^). Oligomeric structures appear to be a common characteristic of BGLs, with the exception of that from *P. horikoshii*. The BGL from the hyperthermophilic bacterium *Thermotoga maritima* forms a dimer (Zechel *et al.*, 2003 ▶), while the BGLs from the hyperthermophiles *Sulfolobus solfataricus* (Aguilar *et al.*, 1997 ▶) and *Thermosphaera aggregans* (Chi *et al.*, 1999 ▶) and BGL A from the mesophile *Clostridium cellulovorans* (Jeng *et al.*, 2011 ▶) form tetramers. BGLPf seems to form a tetrameric structure under physiological conditions in *P. furiosus* cells.

### 3.2. Analysis of the dimer interface of BGLPf   

**Figure 2:**
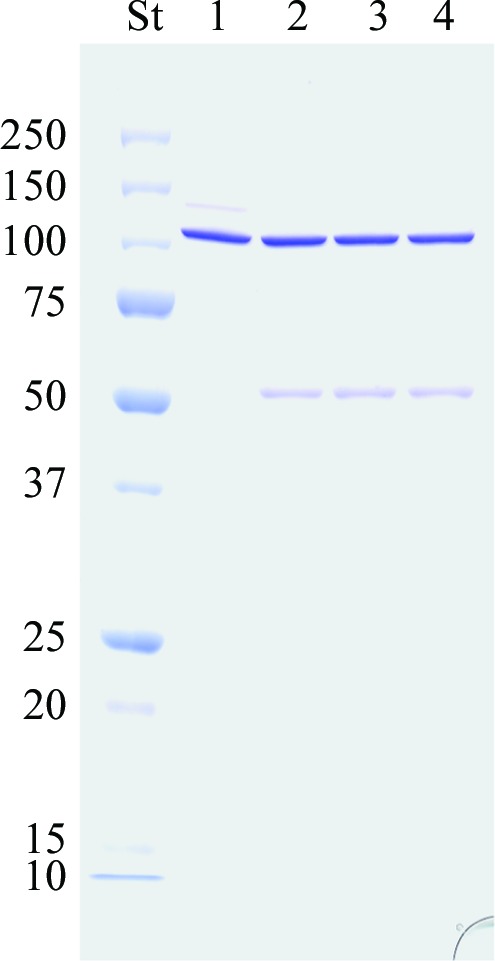
SDS–PAGE of BGLPf. Lane St, relative molecular-weight standards; lane 1, BGLPf prepared in reducing SDS–PAGE loading buffer (2% SDS and 710 m*M* β-­mercaptoethanol) without heating; lanes 2, 3 and 4, BGLPf prepared in reducing SDS–PAGE loading buffer (2% SDS and 710 m*M* β-mercaptoethanol) and heated at 368 K for 5, 10 and 20 min, respectively. The molecular weight of monomeric BGLPf is 54.7 kDa.

The results of reducing SDS–PAGE experiments with BGLPf are presented in Fig. 2 ▶. BGLPf (optimum temperature of about 373 K) in SDS–PAGE loading buffer (consisting of 2% SDS and 710 m*M* β-­mercaptoethanol) migrated with an apparent relative molecular mass of about 110 kDa, which corresponds to the molecular size of a dimer. Interestingly, BGLPf that had previously been heated to 368 K in reducing SDS–PAGE loading buffer also migrated with apparent relative molecular masses of about 110 kDa (major band) and 55 kDa (minor band). These results indicate that most of the dimers in the BGLPf tetramer are hyperthermophilic and stable towards SDS. Similar results have previously been obtained with BGLSs tetramers (Gentile *et al.*, 2002 ▶).

To analyze the dimer interface, the interactions between each monomer of BGLPf, BGLSs and BGLACc were examined using the *Protein Interfaces, Surfaces and Assemblies* (*PISA*) web server (Krissinel & Henrick, 2007 ▶; Table 2 ▶).

In BGLPf, the interfacial contacts within the tetramers were mainly hydrophobic, with some specific polar interactions. The number of salt bridges and hydrogen bonds found in the *A*–*C* interface was lower than that in the *A*–*B* interface. However, the solvent-inaccessible area of the *A*–*C* interface was found to be larger than that of the *A*–*B* interface (Table 2 ▶). The averaged solvation free energy (Δ^*i*^ *G*) for the *A*–*C* interface was also more negative than that for the *A*–*B* interface. Remarkably, the hyperthermophilic *A*–*C* dimer of BGLSs is stable even at 358 K (Moracci *et al.*, 1995 ▶). The *A*–*­C* dimer of BGLSs presents similar solvent-inaccessible area and averaged solvation free-energy values to those of BGLPf and these values are larger than those of the *A*–*B* dimer of BGLSs, which is in good agreement with our results (Gentile *et al.*, 2002 ▶). When comparing BGLPf and BGLACc, both solvent-inaccessible area and averaged solvent free energy in BGLPf were much larger than those in BGLACc. In conclusion, thermostable BGLPf has a comparatively large solvent-inaccessible area at the *A*–*C* interface; the averaged solvation free energy is reduced, thereby providing the *A*–*C* dimer with hyperthermostability. These results suggest that in BGLs the *A*–*­C* dimer is more stable than the *A*–*B* dimer. Furthermore, the hyperthermostability of the tetramer structure of BGLPf seems to mainly be controlled by entropy-driven interactions.

### 3.3. Structural comparison between BGLPf and BGLACc   

**Figure 3:**
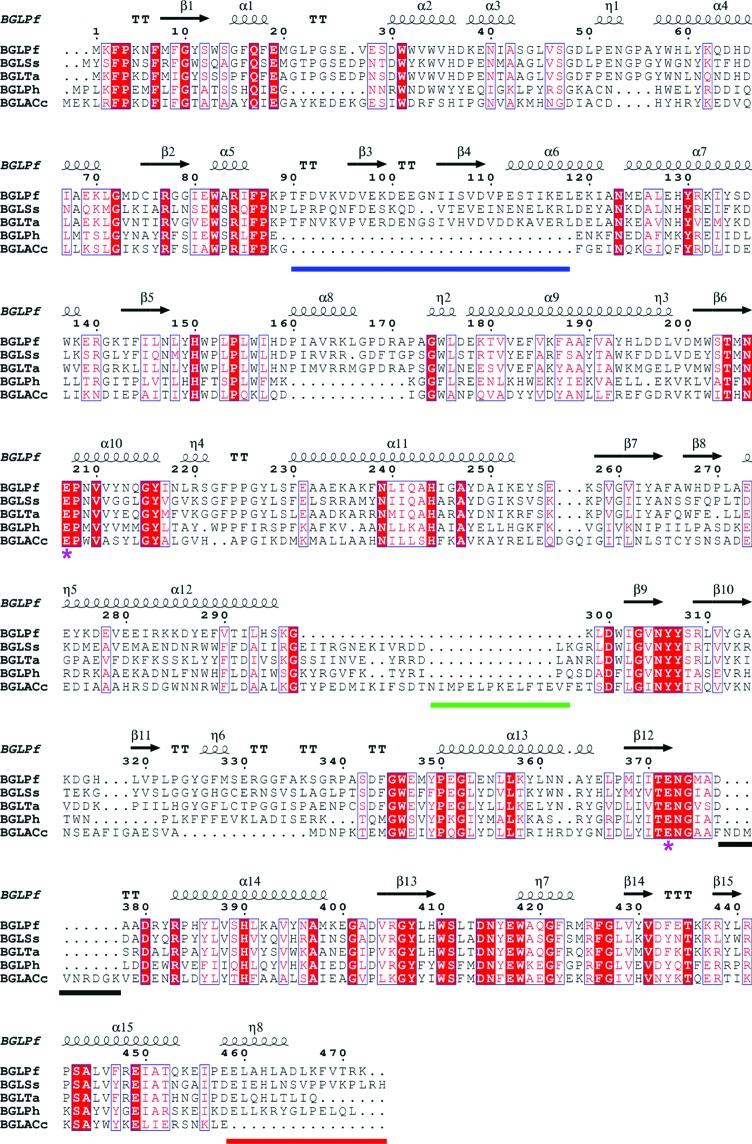
Sequence alignment of archaeal BGLs from *P. furiosus* (BGLPf; PDB entry 3apg), *S. solfataricus* (BGLSs; PDB entry 1gow), *T. aggregans* (BGLTa; PDB entry 1qvb) and *P. horikoshii* (BGLPh; PDB entry 1vff) and mesophilic BGLA from *C. cellulovorans* (BGLACc; PDB entry 3ahx). Residues conserved between BGLs are shown on a red background. Similar residues are shown in red. Similar and identical residues are boxed in blue. Secondary structure is shown above the alignment. Residues in the active centre (Glu207, Glu372) are marked with purple asterisks. The blue, green, red and black lines indicate insertions or deletions. The blue, green and red lines correspond to the coloured circles in Figs. 4 ▶(*a*) and 4 ▶(*b*).

**Figure 4:**
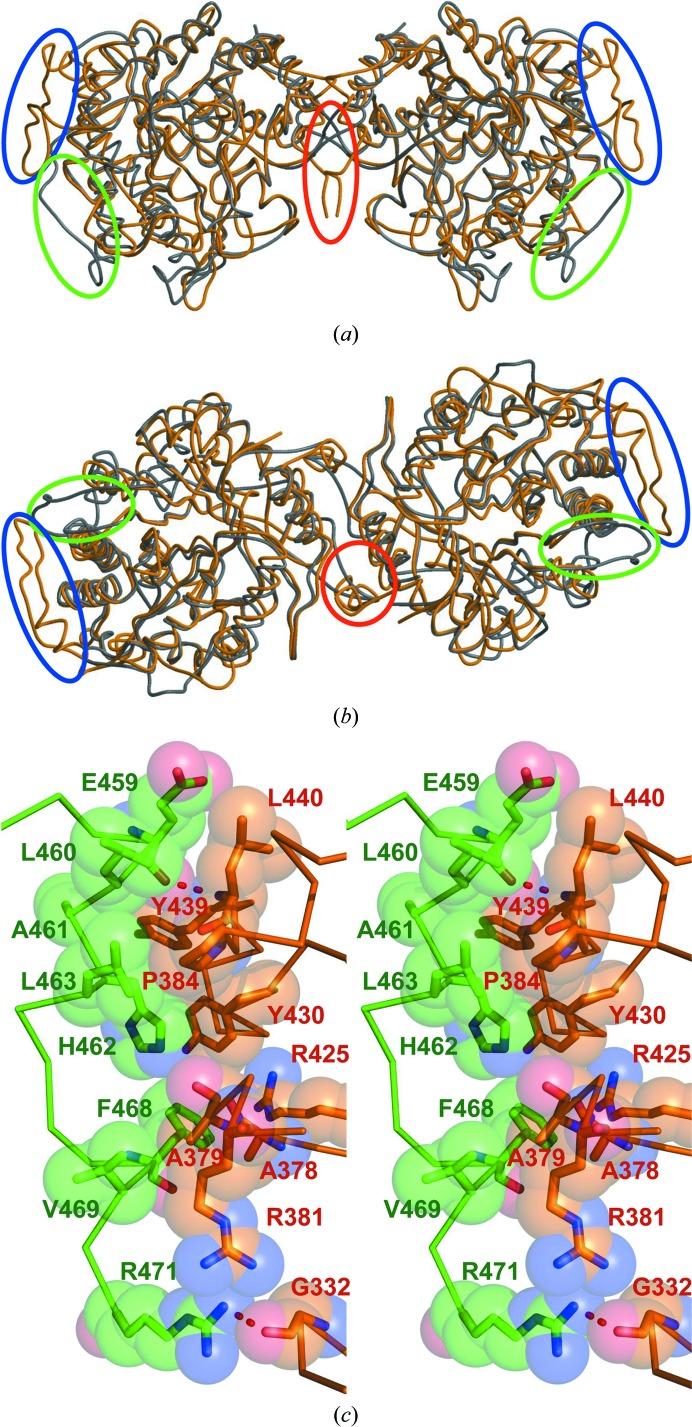
Structural comparison between dimeric BGLPf (orange) and BGLACc (grey). (*a*) Side view, (*b*) top view. The monomer structure of BGLACc was superimposed on each of the protomers in the *A*–*C* dimer of BGLPf. The r.m.s.d. between 278 (molecule *A*) or 279 (molecule *C*) atom pairs was 0.91 Å (molecule *A*) or 0.91 Å (molecule *C*), respectively. Blue, green and red circles show the structural differences between BGLPf and BGLACc, corresponding to each of those coloured lines in Fig. 3 ▶. (*c*) Stereoview at the dimer interface of BGLPf for the C-­terminal part around the orange circle shown in Fig. 4 ▶(*b*). A hydrogen bond is shown by the red dotted line. Detailed information is shown in Table 3 ▶.

Fig. 3 ▶ shows the sequence alignment among BGLs and Figs. 4 ▶(*a*) and 4 ▶(*b*) show the superimposition of the monomer structure of BGLACc with each of the protomers in the *A*–*C* dimer of BGLPf. Compared with the hyperthermophilic BGLPf and the mesophilic BGLACc, three major differences were observed. Firstly, the insertion from Thr90 to Leu118 (blue line in Fig. 3 ▶ and blue circles in Figs. 4 ▶ *a* and 4 ▶ *b*) exists in BGLPf, BGLTa and BGLSs, but not in BGLACc. This insertion was also not found in BGLPh, the specific hyperthermophilic BGL, which exists as a monomer. Conversely, the insertion in BGLACc from Gly297 to Lys298 (green line in Fig. 3 ▶ and green circles in Figs. 4 ▶ *a* and 4 ▶ *b*) was not found in BGLPf. The structures of these insertions are located outside of the *A*–*C* dimer.

Secondly, the C-terminal part (Glu459–Lys472) of BGLPf (red line in Fig. 3 ▶ and red circles in Figs. 4 ▶ *a* and 4 ▶ *b*) is elongated compared with BGLACc. This elongation is also observed in the other archaeal hyperthermophilic BGLs. The C-terminal part formed hydrophobic patches against Ala378, Ala379, Arg381, Pro384, Arg425, Tyr430 and Tyr439 (Fig. 4 ▶ *c* and Table 3 ▶). Along with the hydrophobic interaction, two hydrogen bonds between Leu440 N and Glu459 O and between Gly332 O and Arg471 NH_2_ were also formed.

Thirdly, in BGLACc the hydrophobic interaction found in BGLPf was not constructed owing to the insertion from Asn368 to Lys377 (black line in Fig. 3 ▶). In other words, the insertion of the C-terminal end (red line in Fig. 3 ▶) in archaeal hyperthermophilic BGLs generated the hydrophobic patches stabilizing the *A*–*C* dimer. In particular, the hydrophobic interaction may contribute to entropy in the Gibbs free energy, reducing Δ^*i*^ *G* at high temperature. Introduction of the C-terminal part of BGLPf into any of the other BGLs may explain the hyperthermostability of their tetramer structure.

## Supplementary Material

PDB reference: β-glucosidase, 3apg

Supporting information file. DOI: 10.1107/S1744309111035238/be5175sup1.pdf

## Figures and Tables

**Table 1:** Data-collection and refinement statistics for BGLPf Values in parentheses are for the highest resolution shell.

Data collection	
X-ray source	BL41XU, SPring-8
Space group	*P*4_3_2_1_2
Unit-cell parameters ()	*a* = *b* = 141.96, *c* = 343.01
Wavelength ()	0.9000
Resolution range ()	50.002.35 (2.432.35)
No. of observed reflections	1079285
No. of unique reflections	145450
Completeness (%)	97.9 (90.9)
*I*/(*I*)	17.0 (2.6)
*R*_merge_ †	0.092 (0.326)
Multiplicity	7.6 (3.0)
Refinement	
No. of protein atoms	15448
No. of water molecules	358
No. of glycerol molecules	5
*R* factor‡	0.177
*R*_free_ §	0.223
R.m.s.d. from ideal values	
Bond lengths ()	0.022
Bond angles ()	1.935
*B* factor (Wilson plot) (^2^)	23.82
Average *B* factor (^2^)	
Protein	23.82
Water	22.71
Glycerol	35.71
Ramachandran plot (%)	
Favoured	96.7
Allowed	99.6
PDB code	3apg

† *R*_merge_ = 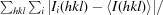 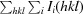, where *I_i_*(*hkl*) are the individual intensities of the *i*th observation of reflection *hkl* and *I*(*hkl*) is the average intensity ofreflection *hkl* with summation over all data.

‡ *R* factor = 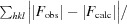 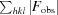, where *F*_obs_ and *F*_calc_ are the observed and calculated structure factors, respectively.

§ *R*_free_ is equivalent to the *R* factor but is calculated for 5% of the reflections chosen at random and omitted from the refinement process.

**Table 2:** Comparison of dimer interfaces and monomers between BGLPf, BGLSs and BGLACc The values for the *A* *C* interaction indicate the average of the *A* *C* and *B* *D* values. The subunits (*A*, *B*, *C* and *D*) of BGLSs and BGLACc correspond to these of BGLPf. 1cal = 4.186J.

	Interaction	BGLPf	BGLSs	BGLACc
Solvent-inaccessible area (dimer interface) (^2^)	*A* *C*	1197.0	1267.2	1003.1
	*A* *B*	940.1	852.9	649.0
Average No. of hydrogen bonds	*A* *C*	6	20	19
	*A* *B*	13	16	3
Average No. of salt bridges	*A* *C*	0	12	9
	*A* *B*	7	8	5
No. of residues in monomer interface (% of total)	*A* *C*	34 (7.2)	34 (6.9)	30 (6.8)
	*A* *B*	24 (5.1)	24 (4.9)	24 (5.3)
Averaged solvation free-energy (*G*) gain on formation of the interface (kcalmol^1^)	*A* *C*	11.2	11.6	0.9
	*A* *B*	5.5	3.3	1.8
Averaged solvation free energy (*G*) for monomer (kcalmol^1^)		509.9	464.1	442.6
Average No. of ordered residues in monomer		471	489	443

**Table 3:** van der Waals interactions in the *A* *C* interface of BGLPf (distance 3.5)

Molecule *A*	Molecule *C*	Distance ()
Gly332CA	Arg471NH2	3.4
Gly332C	Arg471NH2	3.5
Gly332O	Arg471NH2	2.9
Ala379CB	Phe468O	3.5
Ala379O	His385CE1	3.3
Arg381CA	Arg381O	3.4
Arg381CD	Tyr382OH	3.2
Arg381NH1	Tyr382OH	3.0
Arg381NH2	Arg471NH2	3.5
Arg381O	Arg381CA	3.3
Tyr382CE1	Arg381CD	3.4
Tyr382OH	Arg381CD	3.2
	Arg381NH1	3.0
His385CE1	Ala379O	3.4
Arg425NE	Phe468CZ	3.5
Tyr430CD1	His462ND1	3.4
Tyr430CE1	His462CE1	3.5
	His462NE2	3.3
	His462CD2	3.3
Tyr430CZ	His462NE2	3.5
Tyr430CE2	Phe468CE2	3.5
Tyr439CD1	Glu458O	3.4
Leu440N	Glu459O	3.0
Leu440CD1	Glu459CD	3.4
	Glu459OE1	3.5
Glu458O	Tyr439CD1	3.3
Glu459O	Tyr439CB	3.5
	Leu440N	2.9
His462NE2	Tyr430CE1	3.4
His462CD2	Tyr430CD1	3.3
	Tyr430CE1	3.5
Phe468CD1	Ala378CB	3.5
Phe468CE1	Arg425NE	3.5
Arg471NH2	Gly332O	3.2
	Arg381NH2	3.4

## Footnotes

1 Supplementary material has been deposited in the IUCr electronic archive (Reference: BE5175).
