# Supplementary material for: Structure of hyperthermophilic β-glucosidase from Pyrococcus furiosus
Source: Acta Crystallogr Sect F Struct Biol Cryst Commun. 2011 Nov 25;67(Pt 12):1473–9. doi: 10.1107/S1744309111035238 (PMC3232120; doi:10.1107/S1744309111035238)
Supplement: Supplementary file 1 [file f-67-01473-sup1.pdf]

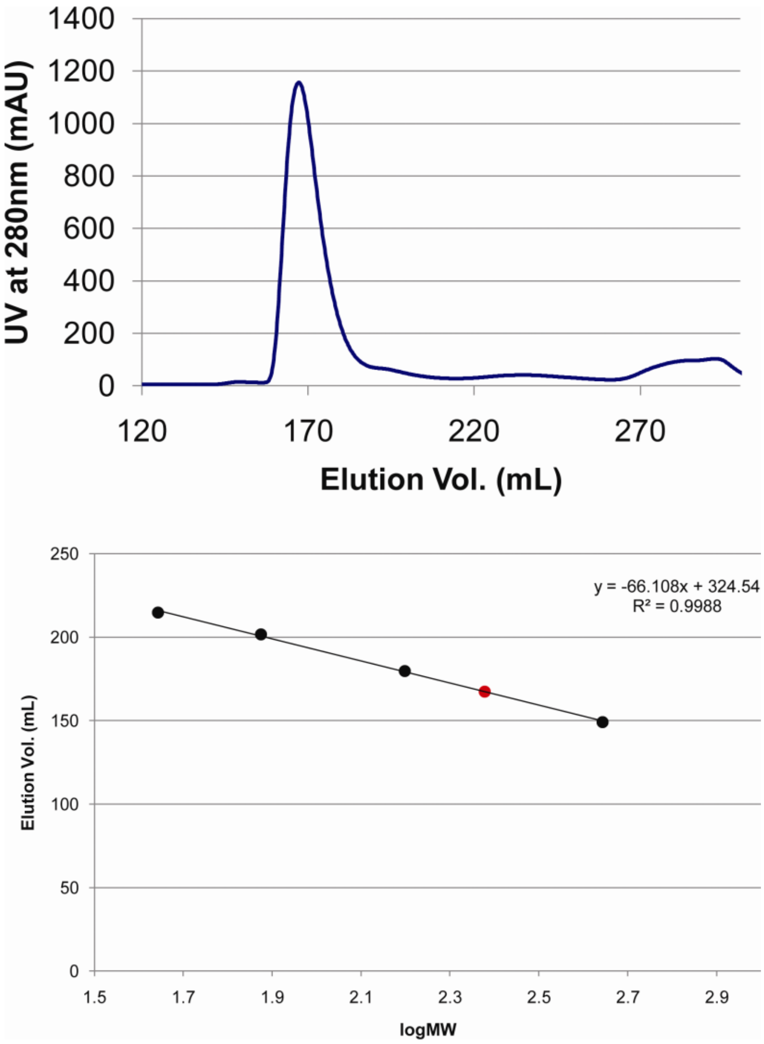

Supplemental Figure 1 (*a*) Gel filtration of BGLPf on Hi-Load 26/60 Superdex 200 pg. Gel filtration of BGLPf gave a single peak. (*b*) Gel filtration of calibration on Hi-Load 26/60 Superdex 200 pg. Standard proteins (ovalbumin, conalbumin, aldolase and ferritin) and BGLPf are shown by black and red plots, respectively. Molecular weight of BGLPf was estimated to be 238.8 kDa.
